# Antimicrobial Stewardship and Urinary Tract Infections

**DOI:** 10.3390/antibiotics3020174

**Published:** 2014-05-05

**Authors:** Lilian M. Abbo, Thomas M. Hooton

**Affiliations:** Department of Medicine, Division of Infectious Diseases, University of Miami Miller School of Medicine, 1120 NW 14th Street, Suite 851, Miami, FL 33136, USA; E-Mail: THooton@med.miami.edu

**Keywords:** antimicrobial stewardship, antimicrobial resistance, UTI, urinary tract infections, antibiotics, duration, appropriate

## Abstract

Urinary tract infections are the most common bacterial infections encountered in ambulatory and long-term care settings in the United States. Urine samples are the largest single category of specimens received by most microbiology laboratories and many such cultures are collected from patients who have no or questionable urinary symptoms. Unfortunately, antimicrobials are often prescribed inappropriately in such patients. Antimicrobial use, whether appropriate or inappropriate, is associated with the selection for antimicrobial-resistant organisms colonizing or infecting the urinary tract. Infections caused by antimicrobial-resistant organisms are associated with higher rates of treatment failures, prolonged hospitalizations, increased costs and mortality. Antimicrobial stewardship consists of avoidance of antimicrobials when appropriate and, when antimicrobials are indicated, use of strategies to optimize the selection, dosing, route of administration, duration and timing of antimicrobial therapy to maximize clinical cure while limiting the unintended consequences of antimicrobial use, including toxicity and selection of resistant microorganisms. This article reviews successful antimicrobial stewardship strategies in the diagnosis and treatment of urinary tract infections.

## 1. Introduction

Urinary tract infections (UTIs) are the most common bacterial infection encountered in ambulatory care settings in the United States, accounting for 8.6 million visits in 2007 [1,2]. Likewise, catheter-associated UTIs are the most common type of healthcare-associated infection reported to the National Healthcare Safety Network (NHSN) [3] and the most commonly treated infections in residents of long-term care facilities (LTCF) each year [4]. In a recent study by Sammon *et al*. [5], 10.8 million patients in the United States visited an Emergency Department (ED) for the treatment of a UTI between 2006 and 2009. The economic burden of utilizing the ED for the treatment of UTIs is estimated to be $2 billion US dollars annually, with mean charges being 10 times higher for patients who were treated and released from EDs ($2000 per visit) compared with treatment in an outpatient clinic ($200) [5].

Starting or reassessing antimicrobial prescriptions based on clinical context, symptomatology and susceptibility data are of paramount importance in all clinical situations and particularly when dealing with UTIs [6]. Urine samples are the largest single category of specimens received by most microbiology laboratories, and the majority of urine cultures do not yield clinically significant results [7]. The diagnosis of UTI is primarily based on signs and symptoms rather than isolated laboratory findings; importantly, bacteriuria is not a disease [8]. Thus, the collection and interpretation of urine cultures should be based on the clinical scenario. Cultures are not recommended for most women with acute uncomplicated cystitis because the microbiology and therapeutic approach for these women is consistent, and short course therapy is effective. However, for individuals with acute pyelonephritis or complicated UTI it is important to obtain a urine culture prior to empiric therapy in order to appropriately tailor the antimicrobial regimen if necessary. In patients with indwelling urinary catheters and residents of long term care facilities, populations with high prevalences of bacteriuria, decisions as to whether to obtain a urine culture and treat should be made carefully in order to avoid inappropriate antimicrobial treatment of bacteriuria that is not associated with symptoms.

Clinicians are frequently faced with the risk-assessment decision to balance the short- and long-term risks and benefits of prescribing an antimicrobial. The short-term risks for the individual prescriber and patient include failure to treat a blossoming symptomatic infection with potential clinical worsening of the patient. The long-term benefits of not prescribing antimicrobials in asymptomatic patients, such as avoiding the emergence of antimicrobial-resistant organisms and adverse events, including *Clostridium difficile* infection, are less tangible to the prescriber focused on the individual rather than the ecological effects with impact at the population level [9].

Antimicrobial resistance is a major public health problem worldwide, caused in part by the overuse of antimicrobials in clinical situations where they are not necessary or in prolonged courses of therapy when shorter durations are as effective [10,11,12]. Antimicrobial prescribing should be prudent, thoughtful and rational. The choice of antimicrobial agents should be individualized based on the patient’s allergy history, local practice patterns, prevalence of resistance, availability, cost and compliance [13]. Unfortunately, in many parts of the world fluoroquinolones are the most commonly prescribed antimicrobials for uncomplicated UTIs even though narrower spectrum cost-effective alternatives are available; their use should be minimized considering their adverse ecologic effects [13]. Several studies in adults and children have demonstrated that short-term antimicrobial courses are as effective as longer ones for the treatment of uncomplicated UTIs and many complicated UTIs [14,15,16], although there still remain many questions as to the optimal duration of treatment for many types of complicated UTIs. It is the responsibility of all healthcare providers to practice antimicrobial stewardship and to avoid the unnecessary use of antimicrobials [17,18].

## 2. Definitions

For the purpose of this review, uncomplicated UTIs include episodes of acute cystitis and pyelonephritis occurring in healthy, non-pregnant, non-immunocompromised women with no history suggestive of an abnormal anatomical or functional urinary tract and no signs of systemic infection. All other UTIs are considered complicated [2]. The classification of UTIs according to the individual host and the severity of location have therapeutic implications in antimicrobial stewardship. International treatment guidelines recommend short-course (single-dose to 3-day regimens) regimens for acute uncomplicated cystitis [13]. Moreover, a Cochrane review [19] of 15 studies, including 1644 elderly women, concluded that short courses (3–6 days) are as effective as long courses (7–14 days) for treating uncomplicated cystitis in elderly women. Short-course regimens are also effective tor treatment of cystitis in pregnant women, UTIs that are generally considered to be “complicated” [20]. Moreover, short-course regimens have been found to be effective for more complicated UTIs—thus, a 5-day course of levofloxacin 750 mg daily was found to be as effective as longer courses of therapy for the treatment of acute pyelonephritis and complicated UTI [15].

Asymptomatic bacteriuria (ASB) is defined as the presence of bacteriuria in urine revealed by quantitative culture in a sample taken from a patient without symptoms suggestive of lower or upper UTI. In women, the traditional quantitative definition for ASB is 10^5^ cfu/mL in 2 consecutive voided urine specimens and for ASB in men a voided urine specimen with 1 bacterial species isolated in a quantitative count of 10^5^ cfu/mL [8]. In general, treatment of ASB is not indicated and may be associated with adverse outcomes, including subsequent antimicrobial resistance, *C. difficile* infection, adverse drug effects, and increased cost. However, ASB is associated with complications in some populations, and should therefore be screened for and treated if present in pregnancy and during interventions that compromise the urinary tract mucosa [21]. Despite the fact that UTI in patients with diabetes mellitus is associated with more severe and uncommon complications, screening for and treatment of ASB in diabetics are not recommended [22].

## 3. Microbiology

*E. coli* causes 75% to 95% of episodes of ASB, cystitis and pyelonephritis in young healthy women, with a minority of cases caused by other Enterobacteriaceae, other Gram negative rods, *Enterococcus faecalis*, *Staphylococcus species* and Group B streptococcus. In men and women with “complicating factors”, the causative uropathogens are more variable.

## 4. Antimicrobial Stewardship Opportunities

### 4.1. Does the Patient Have a UTI? Are Antimicrobials Necessary?

Antimicrobial stewardship opportunities are summarized in Table 1. In general, symptomatic UTIs should be treated with antimicrobials to alleviate symptoms and, in the case of more serious infection, to prevent complications, whereas ASB generally does not warrant treatment. Thus, the first question a clinician should ask when considering antimicrobial therapy is whether the patient is symptomatic and if such signs and symptoms are likely caused by bacteriuria. To assist clinicians in differentiating symptomatic UTIs from ASB, several reviews and consensus guidelines have been published that provide criteria for diagnosis and management of suspected uncomplicated UTIs and those occurring in acute and long term care facilities [2,8,23,24,25,26]. Cystitis is usually manifested as dysuria with or without frequency, urgency, suprapubic pain or hematuria. Clinical signs of pyelonephritis include fever (temperature >38 °C), flank pain, chills, costo-vertebral angle tenderness, and nausea and vomiting [2]. In women, absence of vaginal symptoms in the setting of UTI symptoms increases the likelihood that a UTI is present [23]. It may be very difficult to determine whether symptoms are associated with bacteriuria in patients with altered sensation, such as those with spinal cord injury and neurogenic bladder [25].

**Table 1:** Opportunities for antimicrobial stewardship and urinary tract infections.

Use antimicrobials only when appropriate ASB should be screened for and treated only in select conditions, such as pregnancy and prior to urologic surgery
Use the appropriate antimicrobial Empiric choice—for cystitis, use an agent with low risk of collateral damage For uncomplicated pyelonephritis and complicated UTIs, obtain pre-treatment urine culture and de-escalate as appropriate to narrow spectrum agent
Treat for appropriate duration Use short-course treatment for cystitis Short-course regimens are appropriate for some patients with complicated UTI
Consider non-antimicrobial preventive strategies for recurrent uncomplicated cystitis * Behavioral modification d-mannose Cranberry Topical estrogens in postmenopausal women Probiotics Oral immunostimulants Antimicrobials as a last resort

* Most non-antimicrobial preventive strategies have either not been studied in prospective trials or have not been shown to be effective in trials to date, but are reasonable to try or continue if the patient so chooses and if they are considered to be safe.

Laboratory parameters aid in the diagnosis of UTI but are not helpful in isolation. Furthermore, results of voided midstream urine cultures should be interpreted with caution. In a recent study Hooton *et al*. [27] analyzed microbial species and colony counts in urine samples from 226 healthy women (aged 18–49 years) with symptoms of cystitis. They found that the detection of *E. coli* in voided midstream urine at colony counts as low as 10–10^2^ cfu/mL was highly predictive of its presence in the bladder (positive predictive values of 93% for growth of ≥10^2^ cfu/mL and 99% for ≥10^4^ cfu/mL). On the other hand, growth of enterococcus species and Group B streptococci in voided urine was not predictive of their growth in bladder urine and suggest that these organisms are likely to be urethral contaminants instead. The usefulness of voided urine cultures in other populations has not been studied.

### 4.2. Antimicrobial Selection

Antimicrobial resistance varies over time and by patient population in different geographic locations. If antimicrobial therapy is indicated for UTI, it is important to determine the correct drug, dose and duration of therapy. Sometimes, as with acute uncomplicated cystitis, the clinical presentation is suggestive of a predominant organism (*E. coli*) with predictable antimicrobial susceptibility, and narrow spectrum agents are appropriate for empiric treatment. However, in other situations, as with complicated UTI, antimicrobial susceptibility is not as predictable or there may be multiple causative uropathogens, and broad spectrum agents are more appropriate. The individual risk factors, local patterns of antimicrobial resistance, presence of urinary catheters or other “complicating factors”, recent or prolonged hospitalization and previous exposure to antimicrobials must be taken into consideration when one is considering the optimal empiric agent for treatment of UTI.

Acute uncomplicated cystitis is a benign condition, with early resolution of symptoms in 25% to 42% of women with rare progression to pyelonephritis [2]. Nonetheless, it has considerable morbidity and antimicrobials are routinely prescribed aiming for rapid symptom resolution. The Infectious Diseases Society of America (IDSA) guidelines [13] emphasize the importance of considering collateral damage (adverse effects of a drug, such as selection for resistance) when prescribing antimicrobials. They recommend four agents (nitrofurantoin, trimethoprim-sulfamethoxazole, fosfomycin, and pivmecillinam) that result in relatively little collateral damage compared with other agents. Pivmecillinam may not be available in all countries, which limits its use. Because culture results in these patients are fairly predictable, urine cultures are usually not recommended. However, cultures are recommended if there is a concern about possible antimicrobial resistance since uropathogen resistance data reflected in hospital or community antibiograms are often unreliable, due to the nature of passive surveillance, in guiding the selection of antimicrobial therapy. The IDSA treatment guidelines for uncomplicated cystitis do, however, suggest thresholds for the prevalence of resistance in the community (if reliable antibiogram data are available) above which a drug is not recommended for empiric treatment—10% for fluoroquinolones and 20% for trimethoprim-sulfamethoxazole [2,13].

In addition, pharmacokinetic properties of the antimicrobial are important depending on the site of infection. For the treatment of a complicated UTI, the drug should achieve high concentrations in urine, kidney tissue and prostate. Therefore, nitrofurantoin and fosfomycin are not recommended for upper tract infection or any complicated UTI. Fluoroquinolones have a broad spectrum of activity and penetrate tissue well and are thus the drugs of choice for empiric treatment of uncomplicated pyelonephritis and complicated UTIs. Drug resistance has made this class of antimicrobials less useful than in the past, and for patients with severe infections it is recommended that parenteral agents with more reliable activity against uropathogens be used until susceptibility data are available. Trimethoprim-sulfamethoxazole also penetrates tissue well and is an excellent agent for the treatment of uncomplicated pyelonephritis and complicated UTIs if the organism is known to be susceptible, but it should not be used empirically in such patients due to the high prevalence of resistance among uropathogens worldwide.

### 4.3. Streamlining Empirical Therapy

De-escalation or streamlining a broad-spectrum antimicrobial to a narrower spectrum agent active against the causative uropathogen once susceptibility data are available is an important antimicrobial stewardship strategy in the management of complicated infections occurring in the hospital or LTCF. In addition, selective reporting of antimicrobial susceptibilities for uropathogens is a strategy used in many microbiology laboratories to avoid the use of broad-spectrum agents and guide clinicians in the selection of antimicrobials. The impact of selective reporting on the appropriate use of antimicrobials for UTIs has been evaluated in a randomized study [6] and several prospective surveys [28,29]. Coupat *et al*. [6] randomly assigned residents at 3 French universities to an intervention group that received susceptibility reporting for only 2 to 4 antimicrobials for case-vignettes, or to a control group that received full-length reporting for 25 antimicrobials. Selective reporting improved the appropriateness of antimicrobial choices by 7% to 41%, depending on the vignette. In addition, most residents in the intervention group reported that selective reporting facilitated their choice of antimicrobials. Selective susceptibility reporting has been associated with a direct effect on antimicrobial prescribing by community clinicians in the United Kingdom [29].Tailoring antimicrobial therapy based on local guidelines and culture results and selectively reporting susceptibility for uropathogens are important stewardship practices to improve the appropriate use of antimicrobials.

### 4.4. Selecting the Correct Dose and Route

Pharmacokinetic and pharmacodynamic properties should be considered when treating a UTI in order to achieve optimal tissue levels and effectively eradicate the infection. Antimicrobials that are characterized by concentration-dependent killing (e.g., aminoglycosides and fluoroquinolones) are most effective when administered once daily achieving high serum or tissue peaks relative to the minimum inhibitory concentration (MIC) of the organism. Antimicrobials that are characterized by time-dependent killing (e.g., penicillins and cephalosporins) are most effective when the serum or tissue concentration of the drug is maintained above the MIC for an extended period of time, rather than by achieving high serum concentrations. This is achieved by either continuous infusion or prolonged infusion rates of the antimicrobial. Both types of agents are effective in the treatment of UTI, but it is important that the dose and dosing interval be determined correctly for the agent chosen to treat the infection.

The preferred route of antimicrobial administration depends on the site of infection, antimicrobial susceptibilities, the individual patient’s gastrointestinal absorption and the bioavailability of the drug. Oral agents should achieve high serum and tissue concentrations for the treatment of complicated UTIs. The parenteral route should be used for empiric therapy in severely ill patients or those with poor absorption or oral bioavailability [30,31]. The selection of antimicrobial therapy should also take into account the potential toxicity and necessary dosing adjustments based on the glomerular filtration rate of the individual patient.

### 4.5. Treatment Duration

#### 4.5.1. Cystitis

Recommended antimicrobial regimens and duration of therapy for acute uncomplicated cystitis are summarized in Table 2. For the treatment of uncomplicated cystitis, short-course regimens (single dose to 5 days) are recommended as first-line therapy and are as effective as longer antimicrobial regimens in achieving symptomatic cure with fewer adverse effects [2]. Recommended empirical first-line treatment regimens for uncomplicated cystitis, based on the IDSA guidelines [13], include: nitrofurantoin, trimethoprim-sulfamethoxazole (TMP-SMX), fosfomycin trometamol and pivmecillam. Nitrofurantoin monohydrate macrocrystals 100 mg twice daily with meals for 5 days has shown good efficacy and is well tolerated with low propensity for adverse ecologic effects; it should only be used for the treatment of cystitis and avoided if pyelonephritis is suspected. TMP-SMX 160 mg/800 mg twice daily for 3 days remains very effective with high cure rates [32,33] but is not recommended in areas with resistance prevalence >20% [34,35]; it is inexpensive and well tolerated with fewer ecologic adverse effects than fluoroquinolones. Fosfomycin trometamol 3 g sachet in a single dose or pivmecillinam 400 mg twice daily for 3 to 7 days are also recommended as first line agents due to their low propensity for ecologic adverse effects even though in some studies they appear to be clinically inferior to TMP-SMX or fluoroquinolones [13].

**Table 2:** Antimicrobial management of acute uncomplicated cystitis ^a^.

Antimicrobial	Dosing and Duration	Efficacy
**First-Line Agents**		
Nitrofurantoin monohydrate/macrocrystal ^b^	100 mg twice daily × 5 days (with meals)	Clinical efficacy of 5–7 day regimen: 93% (84%–95%) 3–day regimen appears less effective *vs*. longer regimens Minimal *in vitro* resistance
Trimethoprim-sulfamethoxazole ^c^	160/800 mg twice-daily × 3 days	Clinical efficacy of 3-day TMP-SMX regimen: 93% (90%–100%) Avoid if resistance >20% or exposure in prior 3–6 months
Fosfomycin trometamol	3 g sachet in a single dose	Appears to be less effective *vs*. TMP-SMX or fluoroquinolones Minimal *in vitro* resistance, but most labs do not test
Pivmecillinam	400 mg twice daily × 3–7 days	Clinical efficacy of 3–7 day regimens: 73% (55%–82%) Minimal *in vitro* resistance Unavailable in some countries
**Second-Line Agents**		
Fluoroquinolone: Ciprofloxacin ^c^ Levofloxacin ^c^	250 mg twice daily × 3 days 250 mg or 500 mg once daily × 3 days	Clinical efficacy 90% (85%–98%) High prevalence of *in vitro* resistance in some regions of the world
β-lactam^b^:(e.g., amoxicillin-clavulanate, cefdinir, cefaclor, and cefpodoxime-proxetil)	3–7 days	Clinical efficacy of 3–5 day regimens: 89% (79%–98%) Less effective thanTMP-SMX and fluoroquinolones Prevalence of *E. coli* resistance is variable

Adapted from ref. [2]. ^a^ Efficacy data and antimicrobial recommendations based on IDSA guidelines [13]; ^b^ Pregnancy category B—no clear risk to fetus based on animal and/or human studies; ^c^ Pregnancy category C—animal studies have shown an adverse effect on the fetus; use only if potential benefit justifies the potential risk to the fetus.

Recommended second line agents for acute uncomplicated cystitis include fluoroquinolones (levofloxacin 250 mg or 500 mg once daily for 3 days or ciprofloxacin 250 mg twice daily for 3 days). Due to the rising prevalence of fluoroquinolone resistance in some regions of the world and due to their importance in treatment of a wide variety of infections, the use of fluoroquinolones should be reserved when possible for other uses than cystitis [8,13,35]. Beta-lactams (amoxicillin-clavulanate, cefdinir, cefaclor and cefpodoxime) for 7 days or more are also recommended as second line agents with some studies reporting lower efficacy compared to TMP-SMX and fluoroquinolones [2].

Reducing the duration of treatment and selecting recommended agents other than fluoroquinolones for the treatment of uncomplicated cystitis are important stewardship strategies. Given the ubiquity of cystitis, such stewardship strategies may ultimately have significant beneficial effects on antimicrobial resistance and other adverse consequences of antimicrobial therapy.

Antimicrobial-sparing strategies for the management of acute uncomplicated cystitis that warrant further study include delayed treatment [36] and the use of anti-inflammatory drugs [37].

Treatment duration for complicated cystitis has been less thoroughly studied, but in general such infections should be treated for at least 7 days, especially in men where underlying prostatic infection may exist. Although UTIs in older or pregnant women are often considered “complicated”, short-course treatment has been shown to be effective in such women as mentioned earlier [20].

#### 4.5.2. Pyelonephritis

The treatment of pyelonephritis following initial empiric therapy should be guided by urine culture and susceptibility results. Most episodes of acute uncomplicated pyelonephritis are treated in the outpatient setting, but patients should be hospitalized if the episode is severe, if there is hemodynamic instability, oral medications are not tolerated, poor adherence to therapy or any complicating factors such as diabetes, renal stones or pregnancy [2]. Empiric therapy for pyelonephritis should have a broad-spectrum of activity and be started without delay to avoid complications. For acute uncomplicated pyelonephritis, a fluoroquinolone is recommended as the empiric regimen of choice when feasible [13], because it is a serious infection that may be life threatening. Short-course regimens of oral levofloxacin 750 mg once daily for 5 days appear to be effective for uncomplicated pyelonepritis and complicated UTI [13,15]. Recommended outpatient oral empiric regimens are summarized in Table 3 and include: fluoroquinolones (e.g., levofloxacin 750 mg once daily for 5 days or ciprofloxacin 500 mg twice daily or 1 g extended release daily for 7 days), TMP-SMX 160 mg/800 mg twice daily for 7–14 days or beta-lactams for 10–14 days. A parenteral broad-spectrum agent such as ceftriaxone can be used along with these regimens if drug resistance is a concern, particularly in patients with severe infection [2,13].

Pyelonephritis in patients with “complicating” factors are at greater risk for severe complications. The optimal treatment duration is not known, and such treatment should be tailored to the severity of illness, the rapidity of response to treatment, and results of imaging studies if done. Such patients should generally be treated for 10 days or longer with antimicrobials targeted to the causative uropathogen.

In many studies the optimal duration of treatment for UTIs is defined by the absence of recurrent UTI after an arbitrary number of days (e.g., 7, 10, 14 days). Often, the minimum duration of treatment required for clinical cure is not known. To further reduce volume of consumption, selection pressure and adverse ecological effects, more studies on shorter treatments in different populations are needed [30].

**Table 3:** Antimicrobial outpatient management of acute uncomplicated pyelonephritis ^a^.

Antimicrobial	Dosing and Duration	Efficacy
Fluoroquinolone:		Clinical efficacy of ciprofloxacin 500 mg orally twice daily for 7 days: 96% Clinical efficacy of levofloxacin 750 mg orally or intravenous once daily for 5 days: 86%; *vs*. ciprofloxacin 400 mg intravenous or 500 mg orally twice daily for 10 days: 81%; most subjects in both arms received oral therapy
Ciprofloxacin ^b^	500 mg orally twice-daily or 1 g extended release orally once-daily × 7 days	
Levofloxacin ^b^	750 mg orally once-daily × 5 days	
Trimethoprim-sulfamethoxazole ^b^	160/800 mg orally twice-daily for 14 days	Inferior choice for empirical therapy due to high rates of resistance and corresponding failure rates Highly effective if strain susceptible *E. coli* resistance >20% in many areas of world, including some areas of the US 92% clinical efficacy if *E. coli* susceptible *vs*. 35% if not susceptible)
Oral β-lactam Specific agents are not listed in IDSA guidelines	Duration 10–14 days	Data limited, but inferior efficacy *vs*. TMP-SMX and fluoroquinolones Oral β-lactams should be used only when other recommended agents can’t be used

Adapted from ref. [2]. ^a^ Efficacy data and antimicrobial recommendations based on IDSA guidelines [13]; ^b^ Pregnancy category C—animal studies have shown an adverse effect on the fetus; use only if potential benefit justifies the potential risk to the fetus.

#### 4.5.3. Catheter-Associated UTIs

International guidelines for the management of catheter-associated UTIs (CA-UTIs) [25] recommend 7 days of antimicrobials in patients with prompt resolution of symptoms or 5 days of levofloxacin in patients who are not severely ill (assuming the organism is susceptible). Ten to 14 days of treatment are recommended for patients with a delayed response. A 3-day course of antimicrobial therapy could be used in women ≤65 years without upper urinary tract symptoms after an indwelling catheter has been removed.

### 4.6. Prevention Strategies

#### 4.6.1. Recurrent Acute Uncomplicated Cystitis

Several non-antimicrobial related strategies to prevent recurrent acute uncomplicated UTIs have been published [2]. Behavioral interventions include abstinence or reduction in frequency of sexual intercourse which is often not very feasible. Contraceptive methods such as spermicides and spermicide-coated condoms alter the vaginal flora and favor the colonization of uropathogens and should be avoided. Urination soon after intercourse, drinking fluids, not routinely delaying urination and wiping front to back have not been shown to be associated with a reduced risk of uncomplicated cystitis in case-control studies, but might be effective in some patients and are not unreasonable strategies to suggest for patients with recurrent cystitis. Cranberry juice, capsule or tablets are widely used by women to prevent UTI recurrences, but they have not been convincingly demonstrated to be effective in preventing such recurrences [38]. There are some small studies, however, that suggest cranberry is effective and, given that this strategy appears to be benign, it is reasonable that women continue to use cranberry if they think that it has been effective.

Adhesion blockers such as d-mannose are increasingly being used by women to prevent cystitis, but supportive data are sparse. In a recently published randomized study [39] of 308 women with recurrent UTIs, investigators allocated patients into three groups: 2 grams of d-mannose powder in 200 mL of water daily, 50 mg of daily nitrofurantoin or no treatment for 6 months. Patients in the d-mannose group and nitrofurantoin group had a significantly lower risk of recurrent UTIs during the study compared to patients receiving no prophylaxis (RR 0.239 and 0.335, *p* < 0.0001). Of concern, the authors did not present data for the d-mannose group and the nitrofurantoin group separately, although they mentioned that the difference between the two groups was not significant. Interestingly, the authors noted that the time from starting prophylaxis to onset of symptoms did not differ significantly between the groups (presumably including the no-treatment group). Patients in the d-mannose group had a significantly lower risk of side effects compared to patients in the nitrofurantoin group (RR 0.276, *p* < 0.0001). Porru *et al*. [40], in a recent randomized cross-over pilot trial, evaluated the efficacy of d-mannose in the treatment and prophylaxis of recurrent UTIs in 60 patients (mean age 42 years). Patients were randomly assigned to treatment and prophylaxis with TMP-SMX or to a regimen of oral d-mannose 1 g every 8 h for 2 weeks followed by 1 g twice a day for 22 weeks. Patients were crossed over to the other intervention in the second phase of the study, with no further antimicrobial prophylaxis. Mean time to UTI recurrence was 52.7 days with antimicrobial treatment, and 200 days with d-mannose (*p* < 0.0001). Of note, however, the investigators used an unusual and unproven prophylactic regimen of TMP-SMX in the study (one week per month), observed a highly unusual rate of UTI recurrence in the 24-week period on TMP-SMX (91.7% of women had ≥1 recurrence compared with 20% of the d-mannose women), and the authors do not describe how the data were analyzed for the crossover aspect of the trial. While neither of these studies provide convincing evidence that d-mannose is effective in preventing cystitis, further studies of d-mannose are clearly warranted to determine its pharmacokinetic properties and clinical efficacy.

Other non-antimicrobial strategies to reduce the risk of recurrent uncomplicated cystitis include replacement topical estrogen therapy in postmenopausal women, probiotics, oral immunostimulants and vaccination. Replacement topical estrogen normalizes the vaginal flora in postmenopausal women and has been shown to greatly reduce the risk of recurrent UTI in this population [41]. Probiotics are widely used to prevent recurrent UTI but the published data to date remain unconvincing. Probiotics are touted to protect the vagina from colonization by uropathogens by steric hindrance or blocking potential sites of attachment, production of hydrogen peroxide which is microbicidal to *E. coli* and other uropathogens, maintenance of a low pH, and induction of anti-inflammatory cytokine responses in epithelial cells. However, in a review of four randomized controlled trials of lactobacillus probiotics for bacterial genitourinary infections in women, only one demonstrated a significant reduction in rates of UTI recurrence [42]. Moreover, most of these studies did not determine whether the probiotic led to vaginal colonization with the probiotic strain. While the probiotic approach has a credible scientific basis, additional adequately designed clinical trials need to be performed before its routine use can be recommended. Oral immunostimulants may have a role in UTI prevention. In a systematic review and meta-analysis of four trials that together included 891 participants, OM-89, an extract of 18 different serotypes of heat-killed uropathogenic *E. coli* given orally to stimulate innate immunity, decreased the rate of UTI recurrence (RR 0.61, 95% CI 0.48–0.78) [43]. The agent is commercially available in some European countries but not in the United States. Although there is great interest in developing a safe and effective UTI vaccine, there is no currently available product on the market.

Antimicrobial prevention strategies are highly effective for prevention of recurrent uncomplicated cystitis, but should be considered only as a last resort after non-antimicrobial strategies have been tried or considered and the potential risks of long term antimicrobials have been thoroughly discussed with the patient.

#### 4.6.2. Catheter-Associated UTIs

Screening and treatment of patients with catheter-associated asymptomatic bacteriuria (CA-ASB) are not recommended to reduce subsequent CA-bacteriuria or CA-UTI [24]. Likewise, systemic antimicrobial treatment of ASB is not recommended to reduce the risk of symptomatic UTI in catheterized patients. The most effective way to reduce the incidence of asymptomatic or symptomatic bacteriuria is to reduce urinary catheterization by restricting its use to patients who clearly need it and by removing the catheter as soon as no longer indicated [25]. Nurse- or physician-based electronic reminders and automatic stop orders to remove unnecessary urinary catheters have been successfully implemented in clinical practice and are recommended by the IDSA guidelines. Systemic antimicrobial prophylaxis to prevent symptomatic infection should be avoided in patients with urinary catheterization in order to reduce the selection pressure for multiple-drug-resistant pathogens.

### 4.7. Bacteriuria in Pregnancy

Symptomatic and asymptomatic bacteriuria are common during pregnancy and *E. coli* is the most common etiologic agent. The incidence of ASB in pregnancy varies among different countries and ranges between 2% and 18% [44,45,46]. Studies of ASB have often been of poor quality with small sample sizes, different gestational ages, unclear definitions, differences in diagnostic techniques, timing of urine collection and different cutoff points for significant bacteriuria [47]. In both symptomatic and asymptomatic infection, quantitative culture is the gold standard for diagnosis. Current guidelines recommend screening pregnant women at least once in early pregnancy with a urine culture [22]. Treatment for ASB during pregnancy has become a standard of obstetrical care and has been shown to reduce the rate of pyelonephritis and decrease the incidence of low birth weight. However, studies of ASB in pregnancy were mostly done in the early antimicrobial era and the methodological quality of the studies limits the strength of the conclusions that can be drawn [46]. Duration of therapy for ASB should be 3–7 days [22]. 

There are still unknown consequences of exposing neonates to antimicrobial therapy. A long-term Danish study of 447,629 single pregnancies followed for 9.9 years found a small increased risk of epilepsy in children whose mothers received antimicrobials (mainly for UTI), including nitrofurantoin, during pregnancy [48]. Additionally, there is no clear consensus in the literature on the optimal antimicrobial choice or duration of therapy for UTI during pregnancy. In light of the possible adverse effects of antimicrobials, higher quality research is needed to better understand the direct and indirect consequences of antimicrobial exposure early in life and prudent antimicrobial use is extremely important during pregnancy and early childhood [47]. Studies exploring cost-effective diagnostic tools at the point of care and non-antimicrobial options to prevent or treat ASB and UTIs are needed to limit unnecessary treatment of bacteriuria in pregnancy.

### 4.8. Long Term Care Facilities

One of the most important problems in antimicrobial stewardship in LTCFs is the inappropriate use of antimicrobials to treat UTIs in asymptomatic residents [4,24]. Despite extensive research demonstrating lack of benefit and potential harm for antimicrobial use in ASB [49,50], up to 50% of asymptomatic nursing home residents are prescribed broad-spectrum antimicrobials (e.g., fluoroquinolones) for a suspected UTI [9]. In a study by Phillips *et al*., up to 80% of the antimicrobials prescribed to individuals with an indwelling urinary catheter were written in the absence of signs or symptoms of UTI but in the presence of urinalysis results [9]. The diagnosis of UTIs in elderly LTCF residents is challenging as there is a wide range of events that can prompt urine testing, such as changes in mental status, behaviors, color or smell of the urine with our without dysuria, or falls [51]. Increased antimicrobial stewardship efforts are indicated to reduce unnecessary urinary catheterization, unnecessary diagnostic testing and inappropriate prescribing of antimicrobials for ASB in LTCFs and other institutional settings.

Some useful strategies to improve the use of antimicrobials in LCTFs have been reported [24]. Pettersson *et al*. [52], described an education intervention to improve antimicrobial use in a cluster randomized trial in Swedish LTCFs including educational small group sessions with facility nurses and physicians, guidelines adapted for the local context, written materials, and feedback on prescribing. At the end of the 2 year intervention period, there was no difference between the intervention and control facilities in fluoroquinolone use for UTI. There were, however, significant differences favoring the intervention facilities in secondary outcomes, including a decrease in any antimicrobials given for all infections and an increase in a “wait and see” approach of observation with delayed empiric antimicrobials. Loeb *et al*. [53], in a cluster-randomized trial including 12 nursing homes evaluated the impact of implementation of consensus guidelines with treatment algorithms prior to institution of empiric antimicrobial therapy for treatment of UTIs. The intervention program included nursing education in small group interactive sessions, video tapes and written material, outreach visits and one-on-one physician detailing. Over the study period there was a significant decrease in the number of antimicrobial days given for suspected UTI in the intervention compared with control homes, but no difference between the two groups in total antimicrobial days for all indications. The difference between intervention and control groups appeared to wane over time. Zabarsky *et al*. [54] focused on education of healthcare providers about appropriate collection of urine specimens and not to treat ASB. Direct individual feedback regarding specific cases was given. In the six months following implementation there were significant decreases in the proportion of inappropriate urine specimens sent for culture, episodes of treatment of ASB, and total antimicrobial days. These reductions were maintained during the following 7 to 30 months. Another multicenter study in LTCFs in Finland [55] developed a program where teams comprising an infectious disease consultant, infection control nurse, and geriatrician visited 39 LTCF during 2004–2008. The site visits consisted of a structured interview concerning patients, ongoing antimicrobials, and diagnostic practices for UTI. Following the visits, regional guidelines for prudent use of antimicrobials in LTCFs were published, and the use of antimicrobials was followed up by an annual questionnaire. The investigators found that most of the antimicrobial were used for UTI (range by year, 66.6%–81.1%). At baseline, 14.5% (177/1221) LTCF residents received antimicrobials for UTI prophylaxis and this significantly decreased to 7.8% (90/1158) (*p* < 0.001) after the implementation of the multidisciplinary intervention, without an increase in the number of patients treated for acute UTI in LTCF.

### 4.9. Surgical Prophylaxis

Antimicrobials are often used to prevent specific post-operative infections. Clinical practice guidelines for antimicrobial prophylaxis in surgery [56] from the American Society of Health-System Pharmacists (ASHP), the Infectious Diseases Society of America (IDSA), the Surgical Infection Society (SIS) and the Society for Healthcare Epidemiology of America (SHEA) provide procedure-specific recommendations to avoid post-operative bacteriuria or urosepsis. The selection of prophylactic agents should be based on the individual patient’s prior antimicrobial use, history of UTI and risk factors for UTI. Routine screening and treatment for ASB are discouraged in most surgical procedures as they lead to unnecessary treatment, further diagnostic testing, delays in the procedure, development of antimicrobial resistance and adverse events such as *C. difficile*. Drekonja *et al*. [57], retrospectively evaluated the use of antimicrobial treatment of ASB in 1688 patients undergoing non-urologic procedures at a single center; 25% of the patients were screened by urine culture for ASB. The authors found no difference in surgical site infection rates (20% *vs*. 16%; *p* = 0.56) but more frequent episodes of post-operative UTI (9% *vs*. 2%; *p* = 0.01) among patients treated for bacteriuria *vs*. those not treated. These findings suggest no benefit from empiric peri-operative antimicrobial therapy for ASB.

In urologic procedures such as transrectal biopsy or resection of the prostate, antimicrobial prophylaxis and treatment of bacteriuria is recommended and proven to reduce post-procedural urosepsis from 4.4% to 0.7% [58]. Herr [17] investigated 2010 consecutive patients with bladder tumors who underwent cystoscopy without antimicrobial prophylaxis at a single center by the same surgeon; 24% of the patients had documented ASB prior to the procedure. The incidence of symptomatic post-procedure UTIs within 30 days was 4.5% in colonized patients with ASB and 1.1% in uninfected patients (*p* = 0.02), all UTIs resolved within 24 h with oral antimicrobials. These findings suggest that ASB is common in bladder cancer patients undergoing cystoscopy, but antimicrobial prophylaxis is unnecessary because subsequent UTIs are uncommon and easily treated.

### 4.10. Barriers to Guideline Implementation

Clinicians have profound individual accountability, and yet adherence to guidelines at the bedside often remains low causing omission of therapies contributing to preventable harm, suboptimal outcomes and waste of resources [59]. The reasons for poor compliance with guidelines are multifactorial. Several authors have proposed steps to overcome the barriers in guideline implementation, including more transparency in the level of recommendations, prioritizing which therapies have the greatest benefit to the patients at the lowest risks and costs, and implementation of order sets at the point of care incorporating the recommendations from national guidelines [60,61]. Henig *et al*. [62], systematically evaluated the methodological quality of eight national and international guidelines for the treatment of UTIs in adults published in the last 10 years (2004–2013); the authors identified variable recommendations depending on local epidemiology and different methodological rigor in guideline development. Some limitations to the UTI guidelines include poor descriptions of applicability such as likely barriers and facilitators to implementation, strategies to improve update and resource implications, lack of patient involvement in the development of recommendations and none of the published guidelines used the GRADE methodology to interpret the evidence and grade the recommendations [62]. Existing guidelines for the treatment of UTIs rarely address the implementation of recommendations within antimicrobial stewardship programs.

### 4.11. Areas of Uncertainty

More research is needed to optimize the diagnosis, treatment and prevention strategies of UTIs. Targeted rapid diagnostic tests that could distinguish between inflammation and infection, identify the pathogen and its mechanisms of antimicrobial resistance are very much needed. The development of newer antimicrobial oral agents with novel mechanisms of action against Gram-negative organisms to treat uropathogens is also awaited. Faster diagnostics and better antimicrobials will not improve antimicrobial prescribing practices unless global efforts continue to reinforce the importance of prudent, thoughtful and rational use of antimicrobials.

## 5. Conclusions and Recommendations

The diagnosis of UTI is primarily based on signs and symptoms rather than isolated laboratory findings. Urine cultures are often not useful for acute uncomplicated cystitis, are recommended for patients with uncomplicated pyelonephritis and complicated UTI, and with few exceptions, should not be collected in asymptomatic patients. Antimicrobial therapy should be tailored to each patient taking into consideration the severity of disease, individual and local patterns of antimicrobial resistance and the potential for collateral damage associated with antimicrobial use. Selecting the correct drug, dose, and shortest clinically effective duration of therapy when possible, is key to optimal antimicrobial stewardship. Strategies to prevent recurrent UTIs and catheter-associated bacteriuria could greatly reduce the use of antimicrobials and are therefore key stewardship modalities. It is the responsibility of all healthcare providers to practice antimicrobial stewardship and prescribe antimicrobials prudently, thoughtfully and rationally.
